# Social inequalities in breast cancer screening: evaluating written communications with immigrant Haitian women in Montreal

**DOI:** 10.1186/s12939-020-01322-0

**Published:** 2020-11-23

**Authors:** Marie-France Raynault, Christelle Féthière, Dominique Côté

**Affiliations:** 1 Lea-Roback Research Centre on Social Inequalities in Health, CHUM-Centre hospitalier de l’Université de Montréal, 1301, Sherbrooke East, Montreal, Quebec H2L 1M3 Canada; 2 School of Public Health, University of Montreal, 7101, av. du Parc, Montreal, Quebec H3N 1X9 Canada

**Keywords:** Breast cancer, Mammogram, Screening program, Socioeconomic status, Immigrants, Social inequalities

## Abstract

**Background:**

The province of Quebec (Canada) has implemented a breast cancer screening program to diagnose this cancer at an early stage. The strategy is to refer women 50 to 69 years old for a mammogram every two years by sending an invitation letter that acts as a prescription. Ninety per cent (90%) of deaths due to breast cancer occur in women aged 50 and over. Numerous studies have shown social inequalities in health for most diseases. With breast cancer, a significant paradox arises: its incidence is lower among disadvantaged women and yet, more of them die from this disease. The health care system might play a role in this inequality. The scientific literature documents the potential for creating such inequalities when prevention does not consider equity among social groups. Immigrant women are often disadvantaged. They die of breast cancer more than non-immigrants. Studies attribute this to late-stage diagnosis due to poor adherence to mammography screening programs.

**Purpose of the study:**

The main objective of our research is to assess how Haitian immigrant women in Montreal are reached by the Quebec Breast Cancer Screening Program, and specifically how they perceive the mammogram referral letter sent by the program.

**Methods:**

The study uses a two-step qualitative method: *i)* In-depth interviews with influential community workers to identify the most relevant issues; *ii)* Focus groups with disadvantaged women from Montreal’s Haitian community.

**Results:**

A mammogram referral letter from the Breast Cancer Screening Program may be a barrier to compliance with mammography by underprivileged Haitian women in Montreal. This might be attributable to a low level of literacy, poor knowledge of the disease, and lack of financial resources.

**Conclusion:**

Barriers may be underestimated in underprivileged immigrant and non-immigrant communities. A preventive strategy must be adapted to different sub-groups and must also take into account lower literacy levels. To increase mammography uptake, it is crucial that the benefits of prevention be clearly identified and described in understandable terms. Finally, economic access to follow-up measures should be considered.

## Background

In 2017, it was estimated that there were 26,500 new breast cancer cases in Canada and that 5000 women died of the disease [1]. Ninety per cent (90%) of deaths due to breast cancer in Canada occur in women aged 50 and over [2]. The disease has negative economic impacts not only on family incomes, but also on the state and on the public health system [3]. Indeed, in Quebec, breast cancer ranked fifth in costs for medical services among all types of cancer [4].

To detect the disease early, the Quebec Breast Cancer Screening Program (QBCSP) provides free mammograms to women aged 50 to 69 years old. When they reach age 50, women receive a medical referral letter every two years inviting them to have a mammogram. The strategy is to facilitate medical referrals and monitor test uptake. Since the start of the program in the 1990s, a significant decrease in mortality from breast cancer has been observed [5]. This decline in mortality is due to screening as well as to therapeutic advances. An assessment of the QBCSP’s impact on mortality conducted by the Institut national de santé publique [Quebec national public health institute] showed a 7 to 11% reduction in mortality from breast cancer among women aged 50 to 69 for the years 1998–2003, after considering improved treatments [5].

The QBCSP’s objective is to reduce by 25% mortality attributable to breast cancer over a 10-year period. To achieve this goal, it is important to reach and maintain a participation rate of 70% among the targeted women. However, in 2017, only 56% of Montreal women participated in the program [6]. This is one of the lowest participation rates in the province of Quebec, where the overall rate is 65% [7]. Possible explanations include the low participation of poor and immigrant women [8–11].

The concept of gradient of inequalities in health is not new. In 1980, *Inequalities in Health: Report of a Research Working Group* was published in England [12]. The report, which introduced the concept of inequalities in health, showed that the mortality rate of men at the bottom of the social scale was twice the rate of men in the wealthiest group. Since then, numerous studies [13, 14] have shown that this observation applies to many health issues in different societies, such as incidence of cancers, cardiovascular diseases, and infectious and other diseases. Breast cancer is an interesting case: though its incidence is lower among disadvantaged women, mortality is nevertheless higher among this group [8, 15–17]. In this case, the health care system may play a role in inequality, as screening strategies might be inadequate for these women.

It has been shown that more immigrant women die from breast cancer than non-immigrant women [8, 18, 19]. Interestingly, screening participation rate is lower among immigrants than non-immigrants [9, 20–23]. This might explain part of the inequality in death rates [8, 16]. The scientific literature documents the potential for social inequalities in health when the sole objective of prevention initiatives and health promotion is to improve the health of the population in general, without considering equity among social groups or the particularities of specific social groups [24]. How to reach women of Haitian culture and raise their awareness of the importance of being screened is an important question; it is also one of a global set of questions about outreach strategies for many different cultures, all with potential barriers to getting screened. A study conducted in a metropolitan area showed that 57% of recent immigrants (persons who immigrated less than 10 years ago) did not join the screening program, compared with 26% of non-immigrants [10]. Indeed, there are various cultural barriers to breast cancer screening—not to mention language barriers—in a diverse range of cultures including, for instance, South-American, Asian, Middle Eastern, and African cultures [23, 25–27]. Such barriers include distrust towards medical services [27, 28]; fatalism [27, 28]; stigma associated with cancer [21, 27]; discomfort with the potential of being seen by a male mammography technician or physician [21, 27]; countries of origin whose health systems are focused on treatment rather than prevention [16, 27]; cultures where medical services are sought only when symptoms appear [20, 27]; or cultures where people prefer not to know [27, 28].

In the Saint-Léonard/Saint-Michel district, where 55% of Haitian immigrants to Montreal reside, the participation rate in the QBCSP was 37.7%, the second lowest among the city’s districts [29, 30]. The Haitian community in Montreal is a major one; its 129,000 persons comprise 78% of all Haitians in Canada [31, 32].

Considering the fact more immigrant women die from breast cancer than non-immigrant women [8–10] and that the health care system sometimes plays a role in social inequalities in health, it is important to determine whether the QBCSP outreach strategy and its letter, the focus of this study, might foster this inequality. In cooperation with community partners in the Haitian community and other ethnocultural communities, the QBCSP has used various languages to reach immigrant women via different strategies, including dedicated telephone lines, leaflets and posters, web pages, the involvement of community leaders and health professionals, publicity campaigns, and workshops run in collaboration with community organizations, services, and the media [33]. To our knowledge, no research has tested how the main letter, which is mailed to all women over 50 in Quebec, and is written in French or English, is understood by women from a major ethnic community, the Haitian community.

The main objective of this research was to assess how Haitian immigrant women in Montreal perceive the mammogram screening referral letter sent by the QBCSP. The secondary objective was to evaluate whether the literacy levels of those women play a role in their adherence to the screening program.

## Methods

Qualitative methods were used for the two-step analysis.

First, in-depth interviews were conducted with relevant community workers: professionals from two different local health community centres, and a leading community worker from a Haitian community organization. Interviews were conducted to plan focus groups, determine appropriate questions, and decide on a strategy for addressing the targeted community. The goal of this first step was to understand related cultural phenomena in the Haitian community and address key issues pertaining to the disease, the selected group, and prevention.

Second, focus group participants were recruited via a Haitian community organization. Inclusion criteria were being a Haitian woman aged 40 to 69, and living in a disadvantaged area: in the neighbourhoods of Saint-Michel or Montréal-Nord, which have the lowest literacy levels in Montreal. The participants recruited were either eligible for the QBCSP (50 – 69 years old) or would soon be (40–49 years old). The neighbourhoods targeted also have high concentrations of Haitians [34]. Literacy levels in these areas of Montreal are low. We purposely chose not to investigate or consider participants’ breast cancer history.

Focus groups were conducted to understand women’s personal experiences with, and reactions to, the QBCSP letter [35]. According to Wilkinson (1998), focus groups are a method of choice—particularly among ethnic minority women—to gather data on people’s own meanings of health and illness, and have been used to facilitate access to screening [36].

To encourage dynamic and interactive discussion, each group included 10 to 12 women [37]. On average, the group sessions lasted one and a half hours. The participants’ opinions, feelings, and attitudes about mammograms were documented, as were their understanding of the referral letter and the resulting level of compliance. Focus groups were facilitated by one of the authors who speaks Creole, the language that was used to enhance communication and understanding.

Our research ties in with the CONSORT-Equity 2017 statement [38], which “promotes the reporting of factors that relate to unfair and avoidable differences between population groups” [39] in health intervention effects. We also build on the method used by the CONSORT team to develop this statement: the team drew on patients’ and key informants’ knowledge to improve reporting of intervention effects related to health equity [40]. In our case, the intervention is a screening program, and its effects might differ according to outreach level attained among various population groups.

A plan of topics to discuss was developed, and QDA-Miner qualitative data analysis software was used for inductive analysis of the transcriptions of the focus groups.

### Conceptual framework

**Fig. 1:**
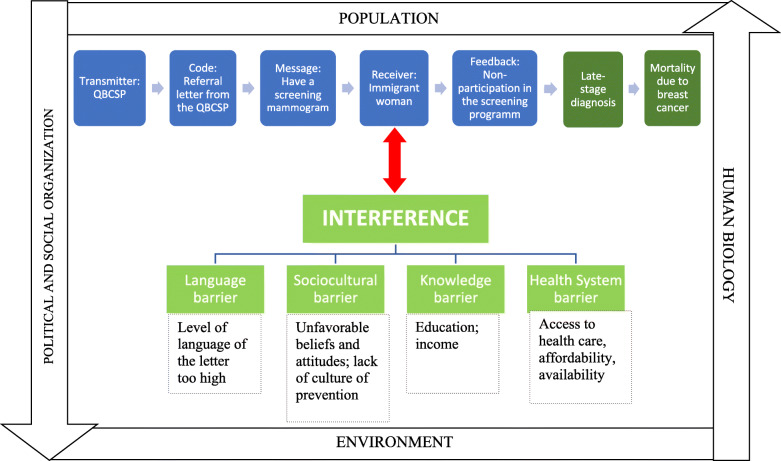
Conceptual framework adapted from cybernetic communication model and social health determinants

We used a conceptual framework (Fig. 1) that integrates both the cybernetic model of communication [41] and health determinants addressed in the literature. The cybernetic model of communication describes transmission of messages in five steps (transmitter, code, message, receiver, feedback). Factors can interfere in message transmission before it gets to the receiver. Here the interfering factors — the barriers to receiving mammograms — are social health determinants.

### Ethics

The project was reviewed by the research ethics committee of the Montreal regional health and social services agency and by the research ethics committee of the University of Montreal. Each participant signed a consent form validated by the committee. The committee also validated the interview guide.

### Setting

Participants expressed their opinions about predetermined themes. Overall, we adopted a non-directive interview style, giving participants flexibility [42] except in cases where their low levels of literacy and education required explanation to get answers. In addition, breakfast or snacks and monetary compensation were offered.

### Data analysis

A thorough analysis of the transcriptions from the three focus groups was performed to determine categories, using the research project’s conceptual framework. We performed an inductive analysis and identified categories pertinent to the objectives of this research [43]. A preliminary classification established the following categories:

- Lack of understanding: Not understanding a question, a concept, or the letter
- Sociocultural characteristics: Expression of beliefs, values, and attitudes for or against adherence to mammography; lack of culture of prevention; income
- Knowledge: Expression of a lack of knowledge or good knowledge of breast cancer or of mammograms
- Health system: Identification of barriers caused by the health system and suggestions for changes to the referral letter

## Results

Preliminary interviews permitted an ideal set-up of the focus groups, better time management, and enhanced participation through the use of Haitian Creole.

Thirty-two women were met. All participants were covered by the provincial government healthcare insurance, which fully covers screening and treatment. After three focus groups, saturation had been reached [44].

Coding resulted in four categories:

1. ***Understanding***
  Not understanding the letter
  Not understanding a question
  Not understanding a word
  Expressing social desirability (respondents conforming their responses to what they assume is socially desirable)
2. ***Sociocultural characteristics***
  Favourable
  Unfavourable
  Mistaken beliefs
  Fatalistic disposition
  Importance of the Creole language
3. ***Knowledge***
  Lack of knowledge
  Presence of knowledge
4. ***Health System***
  Barriers
  Participants’ suggestions for improving the letter

The internal validity of this research was assessed by inter-rater agreement. Verbatim transcriptions were encoded by an external encoder. Overall, the segments were coded the same way by both coders.

Results based on our category definitions are presented here.

### Understanding

#### Not understanding the letter

We observed that level of literacy among the focus groups was low. Indeed, most participants could not read French nor speak the language very well. Participants repeatedly stated they did not understand the letter. One participant, who herself had difficulty reading, read the letter to the others, who could not read.

As explained in the introduction, the letter sent by the QBCSP acts as a referral for a mammogram. However, the participants did not think it looked like a referral form, as intended. They were confused about the contents of the letter. Their questions pointed out the importance of some details omitted from the letter and which need clarification:

‘Do you have to have it again, if you’ve already had one?’

‘What if you’re older than 50?’ [Translation].

#### Not understanding a question

Occasionally, despite the fact that participants were addressed in Creole, their answers were off-topic. This suggests that the question was not properly understood.

The researcher noted moments of silence, again implying lack of understanding. Sometimes, the researcher had to explain, restate, or guide the discussion to ensure that participants understood.

#### Not understanding words

The most misunderstood words were ‘mammogram’ and ‘screening’. However, participants said they understood the word ‘breast’, but would still prefer the Creole translation of the word.

#### Signs of social desirability

There were signs of social desirability when it came to commenting on the health system or the letter. Participants reported being quite satisfied with the letter and had almost no comments on its content and format, despite their lack of understanding. This contradiction will be further discussed below.

### Sociocultural characteristics

#### Sociocultural characteristics favorable to mammography

This category included cultural characteristics specific to Haitians and socioeconomic characteristics attributable to any disadvantaged group.

Women from the Haitian community perceive breasts as a very important part of the female body, representing beauty, pleasure, and maternity. These women are not uncomfortable or inhibited when it comes to breasts.

Haitian women recognize the importance of going to a doctor and having confidence in him or her.

The women said they fear death and feel vulnerable to the disease. We will see below that a minority of participants do not feel vulnerable and are not afraid, because of their fatalistic dispositions.

Participants expressed the importance of having support from the community, especially because they are illiterate:

“Researcher: Do you think that most Haitian women understand the letter?”

“Participants: No. Those who don’t will ask someone to explain it.” [Translation]

#### Unfavorable sociocultural characteristics

As stated previously, some women said they were not afraid of breast cancer:

“I can’t be afraid of the disease since it’s chosen me.”

“You can’t be afraid because it’s not you who put it in yourself.”

“I have children; Jesus will not do this to me.” [Translation].

Participants stated that their friends do not seem to participate in the program.

Finally, a very important social characteristic of these women is using Creole as the language of communication. Participants expressed the need to have a translation of the letter’s key words, so they could understand the main issues presented.

### Knowledge

#### Presence of knowledge

Participants were aware of the disease and had enough knowledge to understand that it is serious. Some referred to the hereditary nature of this disease, mentioning that they would feel more vulnerable if they had a family history of breast cancer. In addition, participants understood that the disease affects all groups, regardless of race, country of origin, or other factors.

As for the causes of breast cancer, some participants said that lifestyle habits like smoking, drinking alcohol, malnutrition, and bad eating habits affected the likelihood of contracting the disease.

Despite having somewhat limited knowledge, and though several women were aware of breast self-examination and knew how to perform it, very few participants were familiar with mammograms.

One participant talked about the reliability of the test. She mentioned the false-positive and false-negative results as being the main reason why she does not get mammograms, along with the anxiety they provoke. This segment was also coded as a barrier.

#### Lack of knowledge

Some participants openly admitted that they know little about the disease:

“My kids are not like me. They know.”

“We know nothing about breast cancer.” [Translation].

There were mistaken beliefs about breast cancer and mammograms. Participants believed that a blow to the breast systematically causes breast cancer. They also thought that mammograms could cause breast cancer because of the pressure on the breasts. Concerns were also raised regarding radiation exposure. There was a misconception about mastectomies as well: participants thought that one cannot function with a missing breast and that cancer will kill no matter what.

Stress concerning the outcome and challenges posed by the disease was also a concern. Participants said they would rather not know they are sick, because knowing would make them sicker.

Participants said that breast cancer is not a prevalent disease in Haiti. In fact, the WHO reports that 6% of all deaths (men and women) in Haiti were attributed to cancer in 2010 [45]. However, participants have known women who had breast problems, but the term ‘cancer’ seems to be rarely used:

“When I lived in Haiti, I didn’t know there was a disease like breast cancer. I saw several women die. When I got here, I understood they probably died from breast cancer. [ …] In my village, in the countryside, not everybody thinks about breast cancer”. [Translation].

Participants were unaware that breast cancer was inside the breast most of the time, and did not necessarily form a lump on the surface. They did not know that breast cancer was an insidious disease that one could not feel.

We noted some confusion between breast and cervical examinations, hence the confusion about mammograms. Participants also did not know the difference between a mammogram and a simple X-ray.

We observed that lack of knowledge was also due to a limited understanding of the French language. As we handed out the QBCSP letter, we observed that the women had difficulty reading and understanding it. It should be noted that in Haiti, French is used by the upper-middle class only.

Another issue was the concept of mail. Many people do not have formal addresses in Haiti. Moreover, in many villages and neighbourhoods, postal service is poor. People in Haiti, particularly in low socioeconomic classes, very rarely receive letters by mail.

### Health system

#### Barriers in the health system

Participants expressed a preference for Haitian doctors so they could communicate in their mother tongue and better express their concerns. They clearly stated their apprehension about not being able to explain their feelings well in French or English.

Other health system barriers noted by participants were not having a family physician, the false positive and false negative controversy (as presented in the news), waiting times in hospitals and clinics, and the cost to avoid waiting lists. Indeed, in Montreal, if a mammogram result is of concern, further exams are prescribed, including ultrasounds. However, waiting lists for these tests can be very long and only patients who can afford it can skip the waiting period and pay to have it in a private clinic.

#### Suggestions for the letter

The QBCSP sends a letter in French or English since Quebec is a bilingual province. To better understand at least the purpose of the letter, participants asked that the words ‘breast’, ‘mammogram’, and ‘cancer’ be translated into Creole.

The letter is one page long and has five dense paragraphs. Participants suggested a shorter letter, which would look more like a traditional prescription, meaning a smaller and less elaborate piece of paper, clearly identified as a prescription referral.

The QBCSP intended to give readers an idea of the size of tumours that can be detected by mammography by drawing a simple dot. Participants did not understand what it referred to at all, and suggested adding a drawing of breasts to illustrate the point and give a better idea of the size of the dot compared to the breast. They also suggested comparing it to the size of a fruit.

Overall, participants felt that the content of the letter lacked appeal. They suggested using simpler, more accessible language and shorter sentences, so that readers are taken straight to the essential information.

Finally, participants suggested promoting mammography and the breast cancer screening program on television and radio during the news because, they claim, all Haitians watch the news.

## Discussion

Study results suggest that the referral letter is an important barrier to getting a mammogram, mainly because it is poorly understood by women with very low literacy levels. The scientific literature also identifies low levels of literacy as an obstacle to mammography [41–53]. The study reveals additional factors influencing compliance with mammography that are related to a lack of understanding of the letter.

Consistent with previous studies, results show that stress related to outcome is a significant barrier to getting a mammogram [10, 54]. According to focus group participants, stress worsens the disease, so they prefer not to know if they have breast cancer. Yet stress, fear, and vulnerability can induce women to get a mammogram since it allows for early detection and saves lives. However, women do not perceive the benefits of screening, an attitude that is not unique to the Haitian culture. It is common in groups with low socioeconomic status [14, 55]. Participants’ friends do not seem to adhere to the program either. This can be attributed to an absence of mutual encouragement from people in their community living in similar conditions. An emphasis on the importance of early detection is crucial to increase adherence to the program. One way to achieve this would be to have a shorter, more focused, and convincing letter.

The in-depth interviews allowed us to understand that women of the Haitian culture sometimes provide an explanation for illness that draws on religious beliefs; disease comes from the devil or from God. Participants seemed to perceive breast cancer as a disease given by God, for reasons beyond their comprehension. Several studies have found that other cultures also believe that the devil can cause a person to develop breast cancer [28, 56, 57]. The devil is also believed to be the cause of other cancers and diseases in Haitian and in other cultures [16, 25, 58–62]. Since mystical and religious beliefs play important roles in Haitian culture, this point should be carefully considered to promote mammograms and increase awareness of breast cancer.

Findings differ from those of other studies on immigrant women and their adherence to mammography [52, 53, 63, 64]. Our results show that women in the Haitian community do not believe that breast cancer only occurs in particular groups of women, but that any woman, regardless of cultural background, could have breast cancer. Therefore, concerns related to stigmatization of groups that are more susceptible to the disease will not need to be taken into account when promoting the program in this community.

Participants’ enthusiasm and their mostly positive comments about the letter were inconsistent and contradicted what is suggested in the literature—that women from disadvantaged backgrounds adhere less to breast cancer screening programs [28, 65–67]. Their responses may have indicated social desirability. It seemed that participants may have wanted to give the impression that they understood everything. One can suggest that this happens in many situations in their daily lives, including at the doctor’s.

Our study addressed several specific points about the content of the letter. First, it indicates that women in the Haitian community are not shy about their breasts, a factor that is favorable for mammography. They requested that the word ‘breasts’ be translated into Creole in the letter so they can better relate to what is being asked. Second, women did not understand the dot used to illustrate the size of a possible tumour detected by a mammogram. To address the issue, it could be judicious to include more explicit drawings of a breast tumour to enhance understanding. Including such drawings would not be a problem, since Haitian women are not shy about their breasts. Third, the fact that most women in focus groups did not know the meaning of the word ‘mammogram’ indicated that there is a mismatch between the language used in the letter and the target community’s literacy levels. Although some women knew what an X-ray is, they did not know that breast X-rays existed, or that a mammogram is an X-ray. Therefore, it would be worthwhile to explain the technical jargon in the letter to make the letter clearer for the women.

In terms of the format of the letter, no participant understood that it was a referral. It is important that it look like a referral for a test, which is usually on a smaller piece of paper, and/or be clearly entitled ‘PRESCRIPTION’. Secondly, it is imperative to be aware that the mail system in Haiti is not the same as in Canada. Women stated that they do not always pay attention to their mail since they are not used to this communication system. Social desirability was present in participants’ responses.

In summary, research results show that there is significant lack of knowledge and understanding of breast cancer and mammograms. Some cultural characteristics can explain the attitudes and beliefs related to this matter. However, a lack of knowledge and understanding as well as negative attitudes and beliefs about mammograms could be countered by introducing better strategies in the health system, especially through the QBCSP referral letter. Moreover, there appears to be a problem with accessibility; the ‘Health System’ category indicates barriers in Quebec’s health care system that cause poor adherence to breast cancer screening and even low utilization of the system. Also, the format of the QBCSP letter and level of language used limit the women’s understanding, which supports our initial hypothesis.

### Strengths

This research approach corresponds to an exploratory perspective. Although the scientific literature acknowledges several barriers to immigrant communities accessing preventive services, this aspect of the QBCSP has never been evaluated.

An important strength of this project is that it was a two-step process: in-depth interviews, notably with leaders of the Haitian community, which permitted optimal preparation of the focus groups; and the focus groups themselves, which were designed in collaboration with the leaders.

The originality of this research project is reflected in the proximity of the researcher to the participants. The fact that she is herself of Haitian descent and speaks Creole enabled her to conduct the focus groups in Creole, be more culturally sensitive, and provide a better interpretation of the results. This facilitated communication and expression of details and feelings that participants were ready to share.

### Limits

Social desirability was repeatedly observed in this study. The researcher asked the questions in such a way as to try to bypass some social desirability biases.

Common limits of focus groups, such as the group effect, were detected. Participants occasionally waited for someone else to respond before contributing their own insights. Use of focus groups probably led to more homogenized results.

The vast majority of participants in this study had problems with literacy. They were unable to understand the letter, which clearly created a barrier to the QBCSP’s objectives. To compensate for this limitation, the researcher read the letter to participants so they could express their lack of understanding. This may have raised additional issues regarding the letter that may not have come up otherwise.

## Conclusions

The main objective of this research project was to evaluate the QBCSP’s strategy of using a referral letter to invite eligible women to have a screening mammogram. The main conclusion is that the technical wording of the letter did not match participants’ literacy levels, which created a major obstacle to adherence to the program. There is an obvious need for simpler wording, as well as a letter in their native language. In addition, participants voiced a clear preference for Haitian physicians, which could enhance adherence to mammography.

To promote a culture of prevention in immigrant communities, there must be a more appropriate communication plan that will make them feel confident and safe. In light of our results, a new referral letter should be prepared and adapted to the targeted community, based on the conclusions of the current study. Qualified Haitian doctors who are waiting to have the right to practice in Quebec could also be involved in promoting participation in the QBCSP. Given the good reputation of these doctors in their country, they could be trained as spokespersons for the breast cancer screening program in their community.

Social inequality in breast cancer mortality could result from a failure of the health system. One might wonder if the referral letter is understood even by native French and English speakers from disadvantaged groups or with low literacy levels. This topic could be the subject of further research.

## Data Availability

The information and consent form used, the data collection instruments, as well as the thorough data analysis process are described and available in Christelle Féthière’s master’s thesis. In agreement with the consent form, recordings of the focus groups and all personal or nominal information on the focus group participants were destroyed and do not exist anymore.
